# Molecular detection and phylogenetic analysis of *Peste des petits ruminants* virus circulating in small ruminants in eastern Amhara region, Ethiopia

**DOI:** 10.1186/s12917-019-1828-6

**Published:** 2019-03-08

**Authors:** Biruk Alemu, Getachew Gari, Geneviève Libeau, Olivier Kwiatek, Menbere Kidane, Rediet Belayneh, Bewuket Siraw, Barbara Wieland, Wondwoson Asfaw, Reta D. Abdi

**Affiliations:** 1 International Livestock Research Institute (ILRI), P.O.Box -5689, Addis Ababa, Ethiopia; 2 Food and Agricultural Organization of the United Nations (FAO), Emergency Center for Transboundary Animal Diseases (ECTAD), Addis Ababa, Ethiopia; 3 CIRAD, Control of Exotic and Emerging Animal Diseases, Montpellier, France; 4 National Animal Health Diagnostic and Investigation Center (NAHDIC), Sebeta, Ethiopia; 5 Tufts University, Agriculture Knowledge, Learning, Documentation and Policy Project, Addis Ababa, Ethiopia; 6 USAID, Livestock Market Development Project AGP-LMD, Addis Ababa, Ethiopia; 7 Department of clinical studies, College of Veterinary Medicine and Agriculture, Addis Ababa University, Bishoftu, Ethiopia; 8 Department of Biomedical Sciences, College of Veterinary Medicine, Long Island University, Greenvale, New York, USA

**Keywords:** PPRV, Small ruminants, Molecular characterization, Isolation, Eastern Amhara

## Abstract

**Background:**

Peste des Petits Ruminants (PPR) is a severe, highly infectious and fatal viral disease of small ruminants. Four lineages of PPR virus have been identified globally based on sequence analysis of the nucleoprotein (N) and fusion (F) gene. The aim of this study was to isolate and genetically characterize recently circulating PPR virus in small ruminants in the eastern Amhara region in Ethiopia. A total of 28 anti-mortem samples (gum debris, nasal and ocular swab) were collected from clinically suspicious animals and examined for the presence of PPRV by a one-step RT-PCR assay. Samples positive with RT-PCR were subjected to isolation of the virus which were subsequently genetically characterized by sequencing of the nucleoprotein (N) gene and phylogenetic analysis of PPR virus (PPRV) strains.

**Results:**

Of the 28 clinical samples examined, 46.4% were positive with RT-PCR for viral nucleic acid. The PPRV was successfully isolated on CHS-20 cell line with the ovine signaling lymphocyte activation molecule (SLAM) receptor expressed on the cell surface and confirmed with RT-PCR and IFAT assay. The nucleotide sequence and phylogenetic analysis indicated that the PPRV obtained were clustered genetically with Lineage IV isolates of the virus.

**Conclusion:**

The successful isolation of the virus and molecular findings of this study confirmed active lineage IV PPRV infections among populations of sheep and goats in eastern Amhara, suggesting risks for potential spread of the disease to currently free areas. Thus, we recommend systematic vaccination to contain outbreaks in affected districts and geographically linked surrounding districts to which the disease could potentially spread due to different epidemiological linkages.

## Background

Peste des Petits Ruminants (PPR) is an acute, highly contagious, trans-boundary and frequently fatal disease of sheep and goats caused by PPR virus, a member of genus morbillivirus of family Paramyxoviridae [1].

Depending on the extent of predisposing factors and the virulence of the virus, PPR severity can be classified as peracute, acute, subacute, and subclinical. The most common form of PPR is the acute form which is characterized by sudden depression, high fever, anorexia, nasal and ocular discharge, mouth erosive lesions, pneumonia and severe diarrhea [2]. The disease mostly occurs in developing countries, particularly in areas where small ruminant farming is an important component of trade and food production [3]. Since 2007, PPR virus (PPRV) has been regarded as an important threat with more than one billion small ruminants in Africa and Asia at risk of infection [4]. PPR is an economically important disease and notifiable the World Animal Health Organization (OIE) due to its potential for rapid spread and associated restrictions on the international trade of animals and animal products [5].

The PPRV genome consists of a single stranded RNA of negative polarity and length of 15, 948 nucleotides [6]. It encodes six structural proteins, the nucleoprotein (N), the phosphoprotein (P), the matrix protein (M), the fusion protein (F), the haemagglutinin protein (H) and the large polymerase protein (L), and two non-structural proteins, V and C. The gene order is 3′-N-P(C/V)-M-F-H-L-5′ [7].

While only one serotype of PPRV has been identified [8], it can be classified into four distinct lineages based on partial sequence analysis of the fusion (F) and nucleoprotein (N) genes, corresponding with geographical distribution of the virus [8–10]. The PPRV isolates of lineage I and II have been reported in Western and Central Africa, lineage III is most prevalent in Eastern Africa and the southern part of the Middle East, whereas, the lineage IV is common in Asia [11, 12]. The spread of Asian lineage IV to Central Africa, North Africa (Morocco, Algeria, Egypt and Tunisia), and northern part of East Africa (East Sudan and Eritrea) has been observed since the mid-2000s [13, 14].

In Ethiopia, the presence of the disease was first suspected in 1977 in a goat herd in the Afar region, in the east of the country based on clinical evidences [15]. The virus was detected in 1994, and subsequently the isolate reported in 1996 was genetically determined to cluster in lineage III [16, 17]. Lineage IV PPRV has been recently reported from a disease outbreak in Ethiopia in 2010 [14]. However, the epidemiological linkages and spread of the PPR strains are not well understood. There is also continuing occurrence of PPR in small ruminants in Ethiopia requiring research in molecular characterization of the spreading virus strains and further phylogenetic analysis. The aims of the present study were to isolate and genetically characterize the phylogenetics of recently circulating PPR virus in small ruminants in the eastern Amhara region in Ethiopia.

## Methods

### Study area

**Fig. 1:**
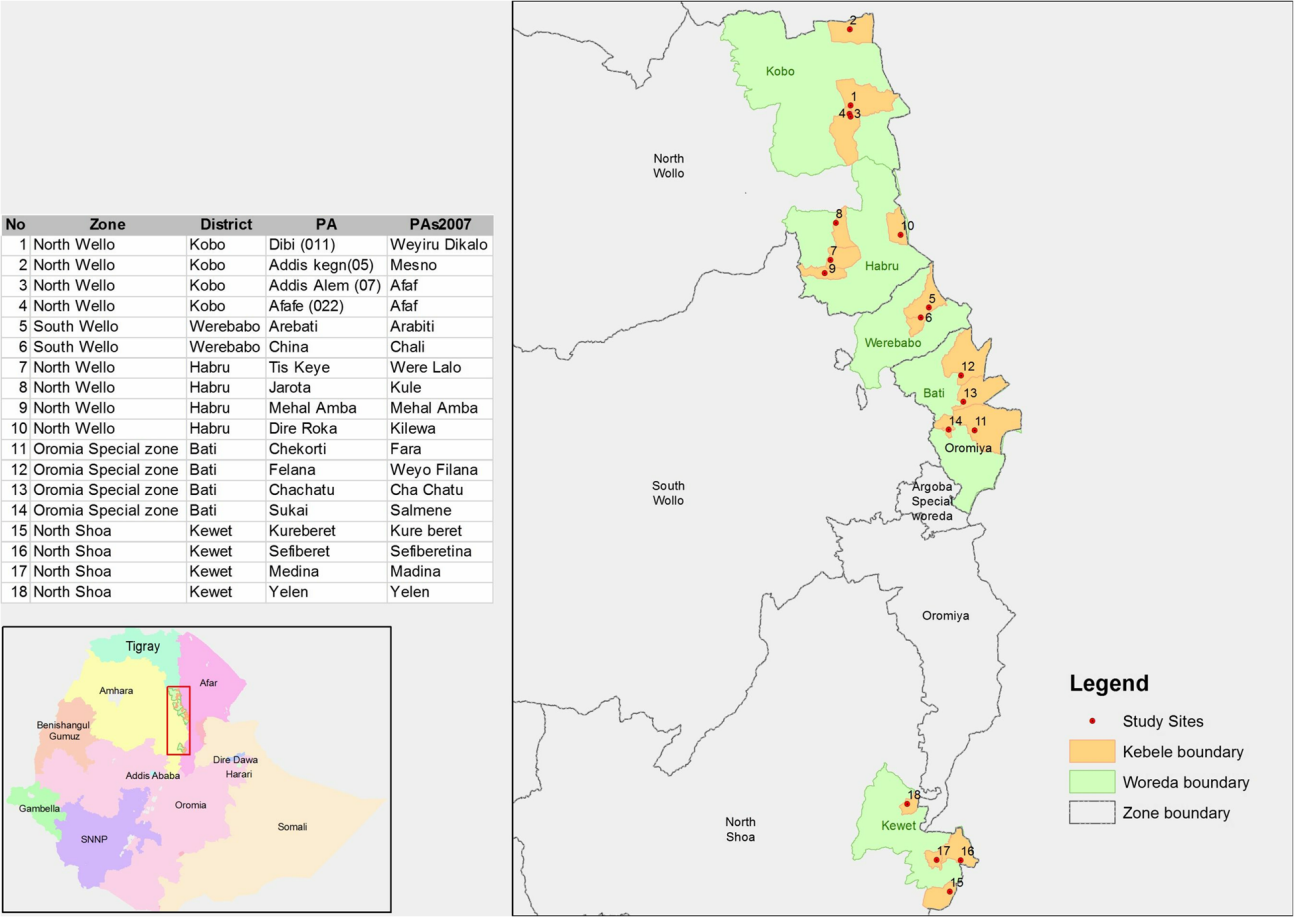
Map of Ethiopia showing regions, study zones, districts and sampling sites. PA: peasant association names used by local community; PAs2007: peasant association names sourced from 2007 census map

The study area purposively targeted Rift valley escarpments of the eastern Amhara region. It includes districts immediately adjacent to the pastoral areas in the Afar region and is epidemiologically closely linked to these areas through seasonal mixture of the herds during grazing and marketing. Previous studies conducted in Afar adjacent districts indicated a high prevalence of PPRV and risk in small ruminants [2, 18–20]. To the north and south, the study region neighbors the highland areas of Tigray and Oromia special zone where PPR outbreaks are rarely reported. The study was combined with a serological survey in 246 households in which 3–4 animals per household were examined. The survey included 18 villages randomly selected from five districts: Kobo and Habru districts from North Wollo zone, Werebabo district from South Wollo zone, Bati district from Oromia special zone and Kewet district from North Shoa zone. The study area map was created using ArcGIS (Fig. 1).

### Field investigation and sample collection

In each village, health status data were collected by recording occurrence of clinical signs that indicated PPR, the number of sick animals observed overall, and number of deaths associated with observed clinical cases were collected.

Of small ruminants with clinical signs suggestive of PPR, nasal, oral, ocular or gum debris swab samples were collected. The samples were collected using sterile swabs which were placed in a viral transport media (VTM) containing PBS, antibiotic and antifungals in a sterile universal tube.

Samples were kept chilled on ice during collection and for shipment to the National Animal Health Diagnostic and Investigation Center for laboratory analysis. They were not frozen before attempting virus isolation but kept chilled at + 4 °C until the analysis done on the next day of their arrival.

### Molecular detection of the virus nucleic acid

The collected samples were examined for the presence of PPRV RNA by the one step reverse transcription- polymerase chain reaction (RT-PCR) assay [21].

RNA extraction from samples was done using a commercial RNA extraction kit (Qiagen® RNeasy Mini Kit, Courtaboeuf, France) as per the manufacturer’s instructions. Reverse Transcription- Polymerase Chain Reaction (RT-PCR) was performed for the N-gene of PPRV using the QIAGEN® one step RT-PCR kit as per the manufacturer’s instructions. The reverse transcription and PCR were carried out sequentially in the same tube. The RNA obtained was converted to cDNA using a reverse transcriptase enzyme. The cDNA was amplified using PPRV specific NP3 and NP4 primers as previously described by [9].

The master mix contained the following reagents: 7 .5μl of RNase-free water, 5 μl of 5X PCR buffer, 1 μl of dNTPS mix (10 mM each), 1 .5μl of each primer; NP3: (5′- GTC TCG GAA ATC GCC TCA CAG ACT - 3′) and NP4: (5′ CCT CCT CCT GGT CCT CCA GAA TCT 3′) at final concentration of 6 μm, 5 μl of Q solution and 1 μl of Qiagen enzyme mix.

The amplification was carried out with the final reaction volume of 25 μl containing 22.5μl of the prepared master mix and 2 .5μl of RNA template. This mixture was submitted to a thermal cycling profile of initial reverse transcription at 50 °C for 30 min, PCR activation at 95 °C for 15 min, followed by 40 cycles of denaturation at 94 °C for 30s, annealing at 60 °C for 30s, extension at 72 °C for 1 min and final extension at 72^o^c for 5 min in an Applied Biosystem 2700/2720 Thermal cycler PCR machine.

Each PCR product (amplicon) of 10 μl were analysed by gel electrophoresis at 120v/80 mA for 60 min on 1.5% of agarose gel in Tris-borate-ETDA buffer. The gel was stained with ethidium bromide and the DNA bands were visualized by UV transilluminator and the image was transferred to a computer.

### Cell culture and virus isolation

The swabs were thoroughly macerated in the transport medium used for collection. The resulting suspension was transferred to a centrifuge tube and centrifuged at 3000–5000 rpm for 20 min. The supernatant was collected and samples taken from one outbreak area or village were pooled together assuming that the same virus would cause the outbreak in the population. Accordingly, four pooled samples were processed for isolation and identification which were originated from four respective different villages existing in three districts.

The cell culture inoculation was performed based on the method of [22] in a cell line of CHS-20. Monolayer cell cultures were inoculated with the pooled samples and inspected daily for evidence of cytopathic effect (CPE). The flask was frozen as soon as the CPE involved about 70% of the cell layer. The presence of the virus in the medium was confirmed by collecting and testing of the cell culture supernatant by RT-PCR and indirect fluorescent antibody test (IFAT).

### Sequencing and phylogenetic analysis

Subsequently, PPRV PCR amplicons were sent to the Control of Exotic and Emerging Animal Diseases Department in CIRAD, Montpellier, France, to analyze the nucleotide sequences based on highly conserved sequences of the nucleoprotein (N) gene. The nucleic acid sequences obtained in this study from PCR products based on NP3-NP4 primers were aligned with the sequence data from PPRV strains present in GenBank. Phylogenetic analysis was performed on the 255 nucleotides located on the 30 end of the N gene of the virus. A phylogenetic analysis of the aligned sequences was performed by the maximum-likelihood (ML) method based on the Tamura–Nei model with gamma distribution of 4. Bootstrap confidence intervals were calculated on 1000 iterations. Gene sequences were aligned using ClustalW of Geneious software, Maximum likelihood analysis was performed and tree drawn using MEGA version 6 [23].

## Results

### Field investigation

From a total of 969 small ruminants examined in the 246 enrolled households in the survey, 28 animals (2.9%) showed typical clinical presentation suggestive of PPR. These animals were from four study districts with most clinical animals (*n* = 13) found in Bati district (Table 2).

**Fig. 2:**
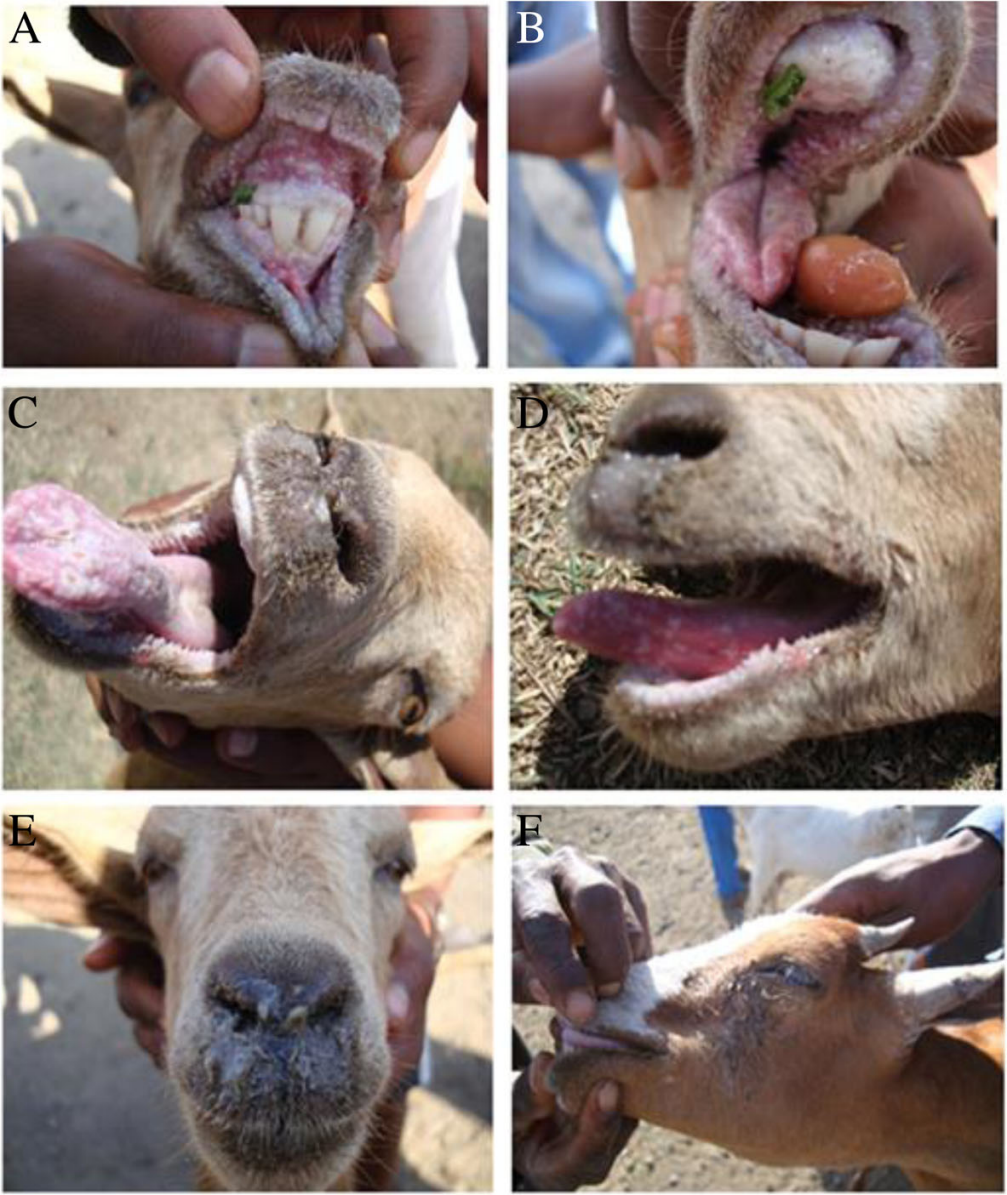
Observed clinical signs of PPR: **a**) Erosive and necrotic stomatitis, **b**) the upper dental pad completely hidden by a thick cheese-like material, **c**) Ulceration on the upper surface of the tongue, **d**) serious nasal discharge, dead cells on the surface of tongue and lesion on lower lip, **e**) muco-purulent nasal discharge, and **f**) lacrimation

Across the five study districts, one severe PPR outbreak was observed in Habru district with animal presenting clinical signs, including nasal discharge, ocular discharge, oral ulcers and nodules, respiratory distress, high fever, abortions and mortality (Fig. 2).

The village affected had 121 sheep and 390 goats and they could be regarded as homogeneous with respect to the risk of transmission of an infectious disease. There were 48 affected sheep and 64 affected goats reported, thus morbidity rates of 39.7 and 16.4%, respectively. Nine sheep and 34 goats had died of the disease resulting in mortality rates of 7.4 and 8.7%, respectively (Table 1). The case fatality rate was 18.8% for sheep and 53.1% for goats. The clinical signs and mortality rate were more severe in goats than in sheep.

**Table 1:** The mortality, morbidity and CFR during a PPR outbreak in Habru district in 2014

Parameters	Sheep	Goat	Total
Population investigated	121	390	511
Morbidity	48 (39.7%)	64 (16.4%)	112 (21.9%)
Mortality	9 (7.4%)	34 (8.7%)	43 (8.4%)
CFR	18.8%	53.1%	38.4%

*CFR* case fatality rate

### Virus detection and confirmation using RT-PCR

**Fig. 3:**
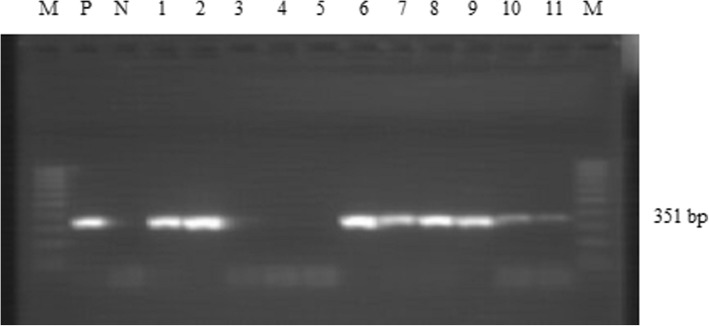
Agarose gel electrophoresis of PCR products (351 bp) amplified with NP3 and NP4, PPR specific primers. Lane M: 100 bp DNA molecular weight marker; Lane P: Positive control; Lane N: Negative control; Lane 1–11: Field samples

From the 28 samples examined with RT-PCR for viral nucleic acid, 13 (46.4%) samples tested positive (Table 2), and as shown in the gel electrophoresis of the PCR products (Fig. 3). The fragment size of the amplified products was 351 bp as reported by Couacy-Hymann and others [9].

**Table 2:** Results of RT-PCR for detection of PPR viral nucleic acid in suspected field samples

Districts	Type of samples	RT-PCR	
		N	Positive (%)
Raya kobo	Nasal swab	4	4 (100)
Habru	Nasal and ocular swab, buccal debris	5	3 (60)
Bati	Nasal swab	13	6 (46.2)
Kewet	Nasal swab	6	0
Over all		28	13 (46.4)

Most virus positive samples were from Raya Kobo district where all 4 samples tested positive and from Habru district where 3 out of 5 samples tested positive with RT-PCR (Table 2).

Similarly, the species-wise comparison in the 28 samples revealed that the PPRV was more often detected in goats than in sheep. The viral nucleic acid was found in 10 out of the 14 goats and in 3 of the 14 sheep. This difference was statistically significant (Table 3).

**Table 3:** Species-wise detection of nucleic acid

Animal species	RT-PCR			
	N	Positive (%)	95% CI	*p*-value
Sheep	14	3 (21.4)	4.7–50.8	0.0229*
Goat	14	10 (71.4)	41.9–91.6	
Total	28	13 (46.4)		

*Fisher exact test

### Virus isolation on CHS-20 cell lines and confirmation by IFAT assay

For isolation and identification, the 13 RT-PCR positive samples were used. They were pooled into four samples, with one pooled sample per village. The PPR virus was successfully isolated on CHS-20 cell lines only from the pooled sample from Tis key village, Habru district (Table 4). On day 1 after inoculation, the CPE was noticed without any succeeding blind passage in CHS-20 cell culture. The appearance of vacuolated syncytia was indicative of CPE in the cell monolayer while no CPE was seen in the control cells. On day 2, the syncytia enlarged to form a large cell clumps that detached from the cell layer. Moreover, the presence of the virus in the infected cell culture supernatant was confirmed using RT-PCR and IFAT assay.

**Table 4:** Summary of RT-PCR positive samples and result of cell culture for pooled samples

District	Village	Species	Sample type	Collection date	RT-PCR	ID Pooled sample	CHS-20
Raya kobo	Addis Alem	Caprine	Nasal swab	15 122,013	+	1	–
Raya kobo	Addis Alem	Caprine	Nasal swab	15 122,013	+		
Raya kobo	Addis Alem	Caprine	Nasal swab	15 122,013	+		
Raya kobo	Addis Alem	Caprine	Nasal swab	15 122,013	+		
Bati	Felana	Ovine	Nasal swab	6 022014	+	2	–
Bati	Felana	Caprine	Nasal swab	6 022014	+		
Bati	Felana	Caprine	Nasal swab	6 022014	+		
Bati	Chachatu	Ovine	Nasal swab	6 022014	+	3	–
Bati	Chachatu	Caprine	Nasal swab	6 022014	+		
Bati	Chachatu	Ovine	Nasal swab	6 022014	+		
Habru	Tis keye	Caprine	Gum debris	29 012014	+	4	+
Habru	Tis keye	Caprine	Ocular swab	29 012014	+		
Habru	Tis keye	Caprine	Nasal swab	29 012014	+		

Negative (−), positive (+)

However, for the remaining three pooled samples four subsequent blind passages were undertaken and the CPE did not develop. The PPRV RNA was not detected from an aliquot of the cells collected at the time of the blind passage from the supernatant medium. The outcomes obtained for all the samples that were tested are summarized in Table 4.

### Phylogenetic analysis

The N gene nucleotide sequences of Ethiopian PPRV PCR amplicons obtained from Habru, Bati and Raya Kobo district were submitted to GenBank and provided with accession numbers KX816962 [Ethiopia_2014_Habru], KX816961 [Ethiopia_2014_Bati] and KX816963 [Ethiopia_2014_Raya_Kobo] respectively.

**Fig. 4:**
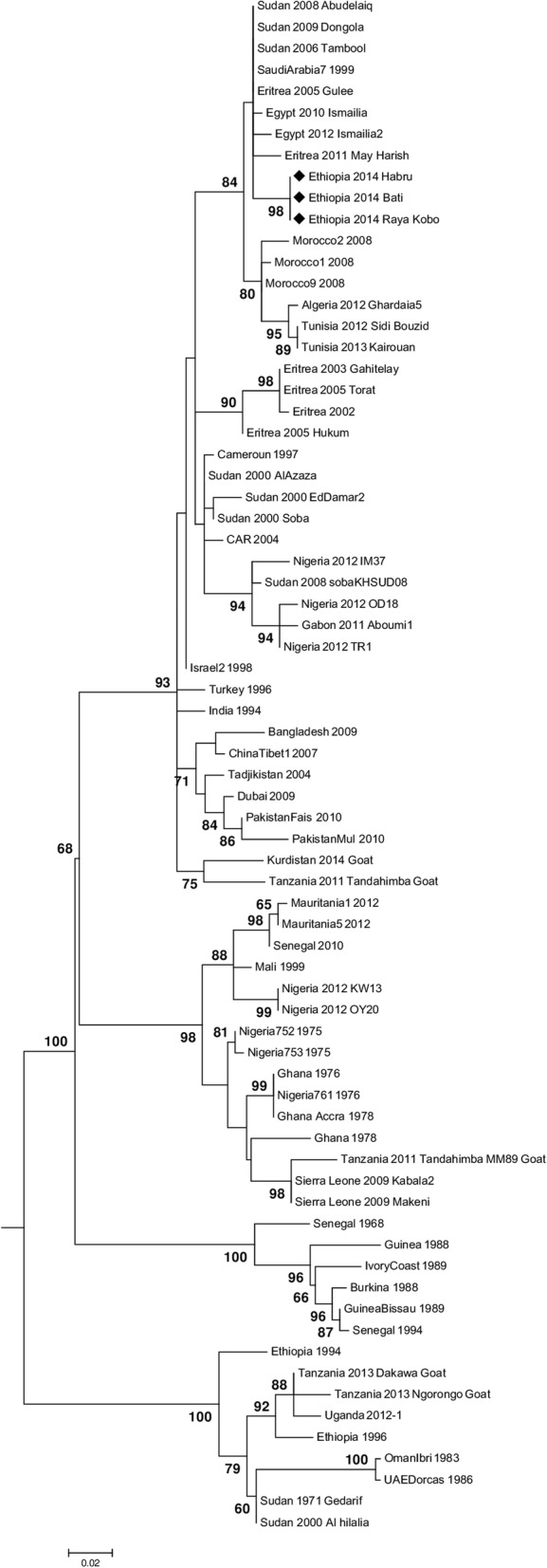
Phylogenetic analysis of nucleotide sequences from the amplified products of PPRV N protein gene with different lineages occurring worldwide

The inferred phylogenetic relationship between the isolates recovered in this study vis-a-vie other PPRVs sequence is shown in (Fig. 4) and that showed that the Ethiopian PPRV isolates belonged to PPRV lineage IV.

## Discussion

Ever since presence of PPR was confirmed in Ethiopia, it has remained a major threat for small ruminant production and has had negative impacts on food security, particularly, in vulnerable regions of the country [19]. Depending on the reported morbidity and mortality and affected flock size and structure of small ruminant production, it is considered as one of the most economically important livestock diseases in some parts of Ethiopia [18].

Although PPR has been a concern for a long time, surprisingly few studies have been undertaken to understand the epidemiology of the disease [2, 18–20, 24] and these studies did not go as far as isolating and sequencing of the isolates. Exceptions are the virus detected in 1994 and an isolate reported in 1996 which was found to cluster within lineage III [16, 17] and the complete genome sequence of a 2010 outbreak isolate [14]. The results of this study provides evidence of the continued spread of lineage IV in Ethiopia and thus a need to further molecular epidemiological studies to understand spread and distribution of different PPRV lineages.

In the present study, PPRV was detected by N gene based RT-PCR in 46.4% of the clinical case samples, which proved the circulation of PPRV in the study districts. However, this might not be a true indication of the PPRV prevalence because only animals showing PPR clinical signs were sampled.

Previous studies in Morocco showed similar PPR positivity rate of 44.4% (16/36) using RT-PCR and higher positivity rate of 80% in Sudan [13]. The presence of PPRV was also confirmed in 33.3% (7/21) and 51.2% (17/33) clinical samples tested in Algeria and north central state of Nigeria, respectively, using a set of primers specific for the F gene of the PPRV [3, 25]. In Northern and Eastern Tanzania, PPRV genome was also detected in 29.6 and 31.1% of the goats tested, respectively [26]. Earlier studies have established that the level of positivity may be influenced by the sample type used during diagnosis of PPR, stage of infection and the type of gene targeted for RT-PCR [27].

The current study revealed a significant higher rate of PPRV infection in goat than sheep samples with RT-PCR. Previously, Abraham and others [18] argued that the apparent absence of pathogenicity in sheep could result from a particular resistance of the local species and/or a loss of virulence of the Ethiopian PPRV strains for sheep. Similarly, Abubakar and others [28] reported that outbreaks of PPR in Pakistan were more severe in goats than in sheep. A higher incidence of PPR infection in goats than sheep was also noted by Mahajan and others [29].

PPR virus should be isolated from field samples in cell culture for further identification, even when the detection of PPR viral antigen has been carried out by rapid immune-capture Enzyme Linked Immunosorbent Assay (Ic-ELISA) [21, 30]. The current study revealed that the inoculation, isolation and propagation of PPR virus in CHS-20 cells was successful from the first passage of one of the four pooled samples, with the CPE characteristic in accordance with that described by the World Organisation for Animal Health [21, 22].

The presence of the virus in the CHS-20 medium was confirmed by collecting and testing of the cell culture supernatant by RT-PCR and IFAT. PPR viral antigen in tissue was detected using FAT [31]. Similarly, the virus was isolated in primary lamb kidney cells and identified by agar gel diffusion testing and Ic-ELISA by [32].

Previously, across both East and North Africa, the circulation of lineage IV PPRV has been reported in Sudan, Eritrea, Uganda, Egypt and Morocco [13, 17, 33]. Lineage IV PPRV had also been recorded from Cameroon in 1997, the Central African Republic (CAR) in 2004 and in Nigeria in 2008. Lineage IV PPRV was reported for the first time in Ethiopia from clinical disease during an outbreak occurred in goats purchased from Debre Zeit market in 2010 [14] and the findings of our study provide evidence that the lineage IV strains has continued to spread in the country as the site from where the sample in this study was collected is about 400 km away from the 2010 outbreak where lineage IV was first showed in Ethiopia. Molecular characterization of circulating strains are thus and important tool to understand the epidemiology of PPRV and track outbreaks in the country. Such information contributes to establishing the diversity and circulation of strains in the field, trace the spatiotemporal origin of a virus, and estimate the risk of its introduction into the herd [33] and may help to characterize eventual differences in virulence of different strains. Also, such insights will help to inform and refine ongoing control and eradication efforts. Therefore, molecular detection and genome sequencing should be included in ongoing surveillance, esp. in active surveillance involving participatory disease search where ongoing outbreaks and cases are found, to allow characterization of the circulating PPRV.

## Conclusions

The successful isolation of the virus and molecular findings of this study confirmed the active PPR virus infections among populations of sheep and goats in eastern Amhara, suggesting risks for potential spread of the disease to currently disease free areas in the country. Thus, we recommend systematic vaccination combined with thorough outbreak investigation and surveillance to contain outbreaks in affected districts. Also recommended are regular vaccination campaigns and strengthening of surveillance systems, with focus on early detection, in epidemiologically closely linked districts to which the disease could potentially spread. Such interventions should be in line with broader regional and national control programs for PPR.

## Abbreviations

cDNA: Complementary Deoxyribonucleic Acid
CIRAD: Center for International Research and Agricultural Development
CPE: Cytopathic Effect
DBARC: Debrebirhan Agricultural Research Center
dNTP: Deoxyribo Nucleotide TriPhosphate
FAO: Food and Agricultural Organization
Ic-ELISA: Immunocapture Enzyme Linked Immunosorbent Assay
IFAT: Indirect Fluorescent Antibody Test
ML: Maximum-likelihood
OIE: World Animal Health Organization
PAs: Peasant Associations
PBS: Phosphate buffered solution
PCR: Polymerase Chain Reaction
PPR: Peste des petits ruminants
PPRV: Peste des petits ruminants Virus
RNA: Ribonucleic Acid
RT-PCR: Reverse Transcription- Polymerase Chain Reaction
SLAM: Signaling Lymphocytic Activation Molecules
UV: Ultra-Violet
VTM: Viral Transport Media
